# Establishing an allergic eczema model employing recombinant house dust mite allergens Der p 1 and Der p 2 in BALB/c mice

**DOI:** 10.1111/exd.12015

**Published:** 2012-10-15

**Authors:** Krisztina Szalai, Tamara Kopp, Anna Lukschal, Caroline Stremnitzer, Julia Wallmann, Philipp Starkl, Luc Vander Elst, Jean-Marie Saint-Remy, Isabella Pali-Schöll, Erika Jensen-Jarolim

**Affiliations:** 1 Comparative Medicine, Messerli Research Institute, University of Veterinary Medicine Vienna, Medical University Vienna, University ViennaVienna, Austria; 2 IPA – Institute of Pathophysiology and Allergy Research, Center of Pathophysiology, Infectiology & Immunology, Medical University of ViennaVienna, Austria; 3 Department of Dermatology, Medical University of ViennaVienna, Austria; 4 Center of Molecular and Vascular Biology, University of LeuvenLeuven, Belgium

**Keywords:** allergic eczema, atopic eczema, Der p1 & Der p 2, house dust mite, protease activity, recombinant allergens

## Abstract

The major house dust mite allergens Der p 1 and Der p 2 are prevalent inducers of eczema. Der p 1 is a cysteine protease disrupting epithelial barriers, whereas Der p 2 functionally mimics the LPS-binding compound MD-2 within the TLR4 complex. In this work, we tested the percutaneous sensitizing capacity of recombinant (r) Der p 1 and Der p 2 in BALB/c mice. Mice were sensitized by percutaneous application of low (10 μg/application) and high dose (100 μg) rDer p 1 or rDer p 2, or with rDer p 1 followed by rDer p 2. Allergen-specific and total IgE antibodies were determined by ELISA. Eczema of BALB/c was classified by the itching score and corresponded to erosions. Infiltrating immune cells were identified by haematoxylin/eosin and Giemsa staining for eosinophils or mast cells, CD3 staining for T lymphocytes. Percutaneous treatments with rDer p 1, but not rDer p 2-induced specific IgG1. However, cotreatment with rDer p 1 led to increase in anti-Der p 2 IgG titres. Both allergens elicited skin erosions because of scratching, thickening of the epidermis, and eosinophil and T-cell infiltration. Our data indicate that recombinant mite allergens in the absence of adjuvant are sufficient for inducing eczema in BALB/c mice. As the enzymatic activity of an allergen might be an important cofactor for specific sensitization via the skin, Der p 1 may act as adjuvant for other allergens too. The presented mouse model is suitable for investigating the mechanisms of allergic eczema.

## Introduction

Atopic eczema (AE), a chronic inflammatory skin disease, is one of the most common symptoms of allergy (1,2). Allergic patients suffering from AE are frequently also affected by symptoms like rhinitis or asthma (3). The clinical hallmarks of AE in humans are dry skin, itching, inflammation, epidermal barrier dysfunction and elevated production of allergen-specific IgE antibodies (4).

The skin reaction in AE can be divided into two phases, an initial acute phase and a second, chronic phase. The immune response of the acute phase is dominated by increased levels of Th2 cytokines, especially overexpression of IL-4, IL-5 and IL-13 (5). Lesional skin is infiltrated by CD4+ T cells, Langerhans cells (LC) and inflammatory dendritic epidermal cells (IDECs) and also eosinophilic granulocytes (4,5). The chronic phase is characterized by a shift to a mixed type of immunological response. In the epidermis, LCs and IDECs with surface bound IgE are dominating, while in the dermis, macrophages are the most notable species of immune cells. Skin lesions in this phase are characterized by elevated mRNA levels for IL-5, GM-CSF, IL-12 and IFN-γ (6,7).

House dust mite (HDM) allergens are important for patients suffering from both allergic asthma (8) and AE (9). More than 30 HDM allergens have been described (10), but most IgE is directed against group I and II allergens (11). These show significant cross-reactivity between the two mite species of highest relevance in Europe, *Dermatophagoides pteronyssinus* and *Dermatophagoides farinae* (12).

In a previous study, we reported the generation of mimotopes, which were applied to define the cross-reactive IgE epitopes of group I and II mite allergens (13). For further proof of concept studies, we considered the establishment of a valid mouse model of importance. Owing to their Th2-biased immune response, BALB/c mice may be considered as a model for mimicking allergic diseases. Therefore, we elucidated the potency of recombinant Der p 1 and Der p 2 in the induction of experimental allergic eczema in BALB/c mice. Matsuoka *et al*. (14) have shown that crude HDM extract applied transdermally was able to induce AE-like skin lesions in mice. In this study, we aimed to go one step further and investigate whether an adjuvant and largely barrier disruption independent animal model based on single recombinant molecules could be established.

## Materials and methods

### Recombinant allergens and animals

Recombinant major HDM allergens Der p 1 (rDer p 1) and Der p 2 (rDer p 2) were produced as previously described in the labs of Prof. Jean-Marie Saint-Remy (15). In short, both allergens were expressed in *Pichia pastoris* and stored in phosphate-buffered saline (PBS). Enzymatic activity was shown for rDer p 1 (16). BALB/c mice (female, 8 weeks old) were purchased from the Institute for Laboratory Animal Science and Genetics (University of Vienna, Himberg, Austria). All experiments were performed according to European Community rules for animal care with the permission number BMWF-66.009/0145-C/GT/2007 of the Austrian Ministry of Science.

### Percutaneous sensitization

**Figure 1:**
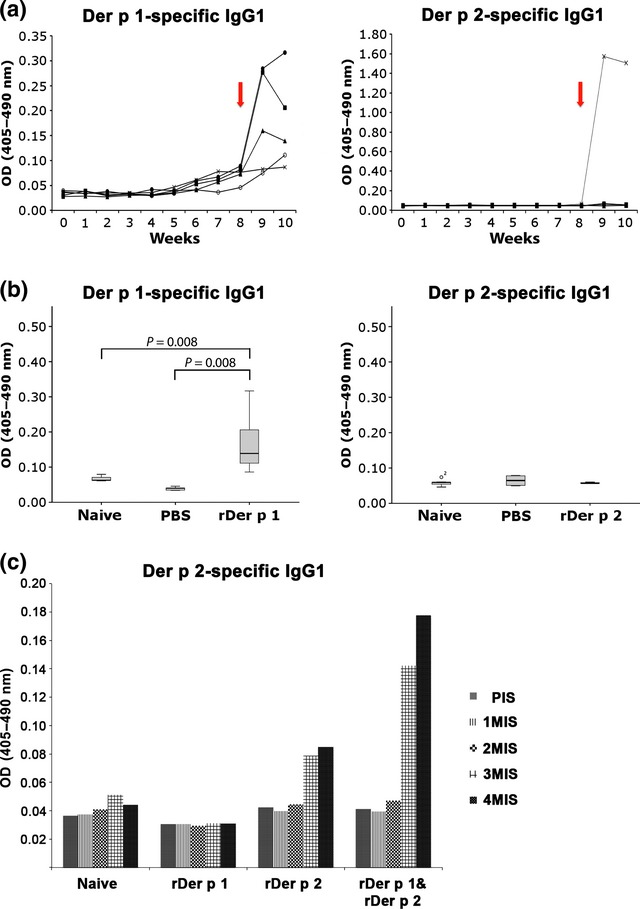
(a) Percutaneous application of 10 μg rDer p 1 for 8 weeks led to low titres of allergen-specific IgG1. After the increase in allergen dose (time point marked by red arrow), treatment with rDer p 1 (left panel showing individual mice) led to the induction of moderate amounts of allergen-specific IgG1 antibodies, whereas only one mouse of the rDer p 2-treated animals produced specific IgG1 (right panel). The treatment scheme is shown in Fig S1a. (b) As compared to naïve and PBS-treated animals, rDer p 1 application resulted in significant induction of specific IgG1 antibodies (*P* = 0.008; left panel), while application of rDer p 2 on the skin failed to induce antibodies with the exception of one animal (OD: 1.8 shown in panel a) but not included in panel b). (c) In a second set of experiment (*n* = 8 per group), an additional group of mice was pretreated for 30 min with rDer p 1 (100 μg) before applying rDer p 2 (100 μg) according to the scheme shown in Fig. S1b. The coapplication with the cysteine protease Der p 1 rendered enhanced induction of anti-Der p 2 IgG levels.

BALB/c mice (*n* = 5/group) were carefully shaved on the back and the recombinant allergens were applied percutaneously with cotton swabs. Mice were carefully wet-shaved on the back using shaving cream. The sensitization was performed 1 day later, using 10 μg rDer p 1 or rDer p 2 in 100 μl PBS for each mouse (14). After assessment of induced antibody levels (Fig. 1a). As negative controls, naïve, shaved-only and shaved/PBS-treated (100 μl PBS) groups of mice were included in the experiment. The mice were treated three times a week on consecutive days for a period of 8 weeks, that is 24 applications in total (Fig. S1a), according to the protocol published by Matsuoka *et al*. (14), allergen dose was increased to 100 μg per application and the sensitization scheme was modified to two applications on consecutive days per week (Fig. S1a).

Blood sampling was performed by tail bleeding before start of sensitizations and afterwards weekly as indicated in Fig. S1a.

In a second set of experiment, BALB/c mice (group size *n* = 8) were shaved as described previously and sensitized percutaneously every third week, altogether three times, with the elevated concentration (100 μg) of rDer p 1 or rDer p 2. In an additional group, mice were pretreated with rDer p 1 (100 μg) and 30 min later rDer p 2 (100 μg) was applied (Fig. S1b).

In this experiment, blood sampling was performed 1 day before the next immunization (Fig. S1b).

### Macroscopic evaluation of skin status

After each administration of allergen or PBS, mice were observed for 15 min and scratching behaviour within the treatment area was documented and evaluated according to the scratching score (0: no scratching; 1: up to 10 strokes; 2: 10–30 strokes; 3: over 30 strokes). All evaluations were performed in a blinded fashion. The skin status was documented photographically at the end of the experiment.

### Analysis of allergen-specific antibodies by enzyme-linked immunosorbent assay (ELISA)

Microtitre plates (Maxisorp, Nunc, Roskilde, Denmark) were coated with rDer p 1 or rDer p 2 (100 μl from 2 μg/ml in 50 mm NaHCO_3_, pH 9.6). After blocking using TBST containing 1% bovine serum albumin (BSA), serum was added diluted 1:100 for IgG1, IgG2a and 1:10 for IgE and incubated overnight at 4°C (100 μl/well). For detection, primary isotype-specific rat anti-mouse antibodies (BD Pharmingen, Schwechat, Austria) diluted 1:700 in TBST/0.1% BSA (RT, 2 h) and secondary peroxidase-labelled anti-rat antibody (GE Healthcare, Buckinghamshire, UK) 1:2000 (RT, 2 h) were used. Detection was performed using ABTS (Sigma, Vienna, Austria), and optical density (OD) was determined using a microplate reader (Spectra Max Plus 384, CA, USA). Statistical analysis was performed using Mann–Whitney test using the software SPSS 14.0 for Windows. Differences were considered statistically at p values <0.05.

### Histological analysis

At the end of the sensitization protocol, mice were sacrificed and skin samples of the treatment area were taken using biopsy punches (kai medical; kai Europe GmbH, Solingen, Germany) from each mouse. Skin samples were fixed in 4% neutral formalin and embedded in paraffin. Paraffin sections were cut to 5 μm thickness using a microtome (Histocom, WR. Neudorf, Austria). Specimens were prepared for staining by deparaffinization (30 min at 60°C, followed by incubation in xylene), rehydration using decreasing ethanol concentration (30 min incubation in total, at decreasing concentration from 100% to 30% stepwise), according to a conventional protocol and tissue reconstitution by 15 min of incubation in PBS. Afterwards, Giemsa and haematoxylin–eosin stainings were performed, the number of mast cells and eosinophils evaluated, respectively, and cell number/mm^2^ calculated.

For immunohistochemical stainings, on deparaffinized skin sections, rehydration and antigen retrieval (with Proteinase K; Roche, Mannheim, Germany) were performed. For the detection of CD3^+^ T cell, after permeabilization (PBS/0.2% Tween20 for 5 min) and blocking (5% FCS/PBS for 30 min at RT) specific CD3-antibody (MCS 1477; AbD Serotec, Duesseldorf, Germany) was diluted 1:50 in 0.1% BSA/PBS and incubated ON at 4°C. On the next day, after washing, secondary antibody goat anti-rat (AF568) was diluted 1:1000 in PBS and incubated for 60 min at RT. After final washing steps, slides were mounted with fluoromount and analysed by light microscope and quantification was performed using TissueQuest® cell analysis software from TissueGnostics, Vienna, Austria.

## Results

### Monitoring the induction of allergen-specific antibodies and total IgE

For the induction of allergic eczema in BALB/c mice, we adapted the sensitization protocol of Matsuoka *et al*. (14). Instead of whole HDM extract, 10 μg rDer p 1 or rDer p 2 were applied to the animals thrice weekly (Fig. S1a). We tested the immunological status of the animals by detecting allergen-specific serum antibodies by ELISA. After 8 weeks, rDer p 1-treated mice already exhibited low levels of antigen-specific IgG1 antibodies (Fig. 1a). In contrast, treatment with rDer p 2 did not induce detectable levels of specific antibodies. Thereafter, we increased the amount of applied allergen to 100 μg (twice a week on consecutive days for 2 weeks) (Fig. S1a). This amended regimen immediately induced allergen-specific IgG1 in all mice of the Der p 1 group, but only in one of the rDer p 2-treated animals (see Fig. 1a,b). Control groups (naïve and shaved/PBS treated) showed no induction of specific antibodies. Neither allergen-specific IgE nor IgG2a could be detected in any group (data not shown), and also no significant difference between treated and non-treated groups in respect to total IgE levels could be observed (data not shown).

### Evaluation of scratching behaviour and skin status after percutaneous sensitization

**Figure 2:**
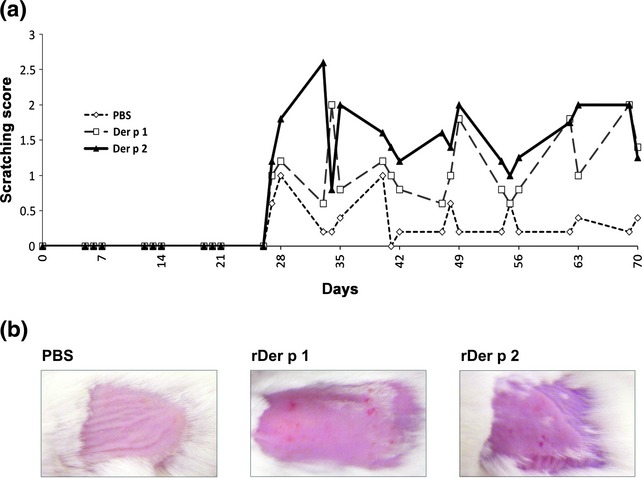
(a) Treatment of animals in both allergen groups led to strongly increased scratching behaviour beginning at week 4 of low dosage treatment, while PBS-treated animals only showed mild reactions limited to occasionally increased scratching in individual animals (dotted line). The graph shows mean scratching behaviour of the three treatment groups assessed for 15 min after treatment and classified according to scratching intensity (0: no scratching; 1: <10 strokes; 2: 10–30 strokes; 3: >30 strokes). (b) The itching-associated scratching behaviour in allergen-treated mice led to skin erosions at the application area, while animals treated with PBS (buffer) were not affected.

After 4–5 weeks, we noted increased scratching behaviour upon percutaneous allergen application. Symptom severity was scored according to the number of strokes observed during the observation period. The allergen-treated groups reached scores up to 2.0–2.5. The shaved/PBS-treated groups showed an overall milder and more homogenous reaction (Fig. 2a).

At the endpoint of the experiment, skin erosions because of scratching and local inflammation were noted in animals treated with either rDer p 1 or rDer p 2. Figure 2b shows a representative photograph from each group. Shaving and application of vehicle alone using cotton swabs did not lead to skin lesions as shown in the example of shaved/PBS-treated mice.

### Histological staining for the identification of infiltrating immune cells

**Figure 3:**
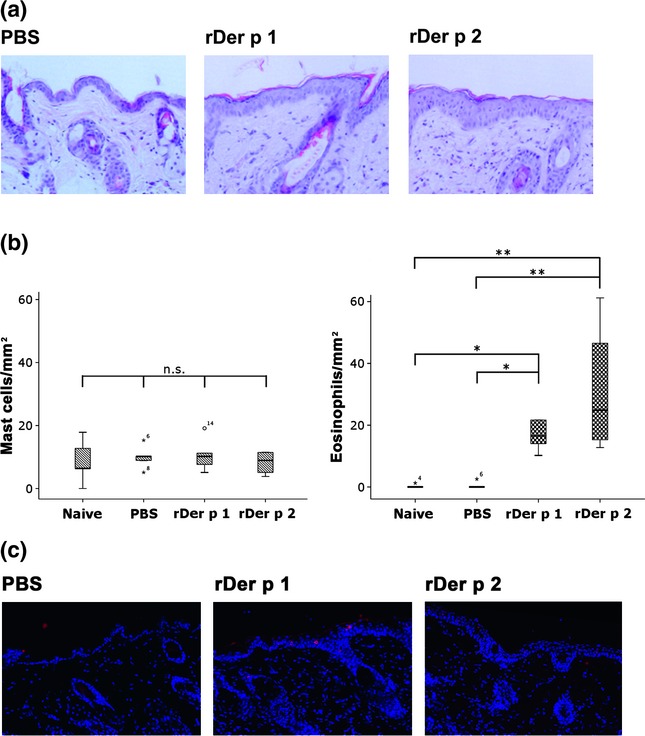
(a) In contrast to PBS-treated mice, animals treated with either rDer p 1 or rDer p 2 showed an increase in epidermal layers and more infiltrates of immune cells into the dermis. (b) The number of mast cells/mm^2^ did not differ between treatment groups (n.s.: not significant), the number of eosinophils was significantly elevated in groups treated either with rDer p 1 or rDer p 2 (*P < 0.05; **P < 0.001). (c) Immunhistochemical staining for CD3 was performed on deparaffinized skin biopsy sections of mice. After primary CD3-specific antibody, goat anti-rat AF568 antibody was used. Finally, slides were mounted with fluoromount and analysed by light microscope; quantification was calculated using TissueQuest cell analysis software from TissueGnostics. The two groups, treated with rDer p 1 or rDer p 2 showed enhanced invasion of CD3^+^ T cells into the epidermis, compared with control.

Skin histology was analysed in biopsies of the treated area of each mouse. Specimens were paraffin embedded and after conventional preparation mast cells were identified by the analysis of Giemsa-stained sections, while eosinophils were detected in haematoxylin–eosin-stained sections. The number of epidermal layers was elevated in BALB/c mice treated with recombinant allergens (see Fig. 3a), while the skin of naïve or shaved/PBS-treated mice remained unaffected (summary of data in Table 1). Analysing the infiltrating cells in the dermis, the number of mast cells did not differ between groups. However, allergen application significantly increased eosinophil counts in comparison with the naïve group and also to the PBS-treated animals (*P* = 0.008 for rDer p 1; *P* = 0.016 for rDer p 2; Fig. 3b).

**Table 1:** Summary of histological skin status after 10 weeks of treatment

Treatment	Epidermal thickening	Dermis (infiltrates)	T-cells (% cells epidermis)
Naïve	None	−	−
Shaved	None	−	−
p.c. PBS	None	−	−
p.c. rDer p 1			
1	6 layers	+++	n.d.
2	None	−	8.5
3	3–4 layers	+++	6.8
4	3 layers	++	4.6
5	3 layers	++	6.3
p.c. rDer p 2			
1	3 layers	++	4.9
2	3 layers	++	5.1
3	6 layers	+++++	2.5
4	5 layers	++++	4.7
5	n.d.	n.d.	n.d.

n.d., not determined.

In immunohistochemistry, enhanced invasion of CD3-positive T cells into the epidermis was observed in the two allergen-treated groups but not in any of the controls (Fig. 3c).

### Adjuvant function of cysteine protease Der p 1 on Der p 2 allergen

In a second set of experiments, mice were sensitized with the higher concentration of the allergen (100 μg/sensitization) and an additional group was added to examine the adjuvant effect of cysteine protease Der p 1 on the sensitization capacity of other HDM allergens, in this case Der p 2 (Fig. S1b). Therefore, in this group, mice were pretreated with rDer p 1, 30 min before further percutaneous treatment with rDer p 2. As shown in Fig. 1c, percutaneous pretreatment with cysteine protease rDer p 1 supported sensitization to subsequently applied rDer p 2, rendering Der p 2-specific IgG1.

## Discussion

Patients suffering from HDM allergy often show symptoms of AE (9), an inflammatory skin disease. Although the pathophysiological mechanism is complex in AE, it is clear that the two major HDM allergens, Der p 1 and Der p 2 are responsible for the majority of IgE reactivity in most of the patients (11).

Most of the published animal models of AE involve some artificial barrier disruption, like for example tape stripping (14),(17), treatment with irritants like SDS (18) or enterotoxin (19), or covering of the treatment area with bandages for extensive periods of time (20). Other studies turn to NC/Nga mice which develop spontaneous AE (21) and demonstrate the impact of exercise-induced stress on AE (22) or the beneficial effect of oregonin treatment (23) on AE symptoms. Also in human patients, AE has in part skin barrier-based pathogenesis. Matsuoka *et al*. 2003 were the first to establish a murine AE model using crude HDM extract without addition of adjuvants. In the present study, we aimed to go one step further and investigate whether an adjuvant and largely barrier disruption independent animal model based on single recombinant molecules could be established. We intended to investigate the influence of protein function and characteristics of the individual allergens using the major HDM allergens Der p 1 and Der p 2 in their recombinant form. In contrast thus to previous work by Yasue *et al*. (24) who applied Der f 1 and Der f 2 intraperitoneally, we aimed to mimic the natural route of allergen encounter.

After 8 weeks of sensitization of BALB/c mice with 10 μg allergen per dose, when testing for allergen-specific Th2 antibodies like IgG1 or IgE, we found that only treatment with rDer p 1 induced moderate amounts of IgG1 (Fig. 1a). Increasing the concentration of the sensitizing allergens to 100 μg/application led to the induction of specific IgG1 in all rDer p 1 treated mice, but only in one animal in the Der p 2 group (Fig. 1a,b). In contrast to Matsuoka *et al*. 2003 who had used crude mite extract in their model for sensitization, we could hardly detect any specific IgE or changes in total IgE levels. This may have to do with the adjuvant function of whole HDM extract as opposed to the here used single recombinant allergens. Nevertheless, increased scratching behaviour compared with the human situation (5) (Fig. 2a) and development of skin erosions (Fig. 2b) was noted in all animals treated with either of the recombinant allergens. In contrast to the aforementioned study (14), examination of skin biopsies revealed the typical patterns of AE-related histological changes, with epidermal thickening, leucocyte infiltrates, including T lymphocytes, in mice treated with rDer p 1 or rDer p 2 (Fig. 3, Table). As shaving combined with sham (PBS) treatment alone did not reveal these pathophysiological changes, we propose that they are linked to the allergen applications. However, we have to admit that wet-shaving might slightly impact the skin barrier even though histopathological changes were missing. Like Matsuoka *et al*., (14) we could not detect any changes of mast cell counts in the dermis of the BALB/c mice; however, infiltrations dominated by eosinophilic granulocytes were observed.

Beside their allergenicity, the major HDM allergens possess additional activities: Der p 1 is a cysteine protease which has been implicated as a Th2 adjuvant in asthma (25). By activating protease-activated receptor 2 (Par-2) on keratinocytes, Der p 1 contributes to skin barrier disruption (26). Group II HDM allergens like Der p 2 are not proteolytically active. They have been suggested to functionally mimic MD-2, a component of the toll-like receptor 4 (TLR-4) sensing LPS (27). Der p 2 was also shown to induce inflammatory cytokines in bronchial epithelial cells (28). In our model, Der p 2 did not induce specific immune reactions, as no specific antibodies could be found (except in one individual probably caused by accidental barrier disruption during the treatment, for instance by scratching). By a combined approach where first Der p 1 was applied to the skin before Der p 2, we could however demonstrate that IgG1 towards Der p 2 can be induced (Fig. 1c). These data are suggestive that Der p 1 with its cysteine protease function may act as adjuvant for Der p 2 and other mite allergens by opening the skin barrier and supporting allergen penetration.

Although there was hardly any IgE and no strong IgG1 antibody response either, to rDer p 1 and rDer p 2 in our model, both allergens elicited itching, eosinophilia and thickening of the epidermis in the animals, which was not caused by mechanical irritation during treatment. In accordance with the “epimmunome” principle (29) we speculate that the intrinsic protein characteristics of the allergens elicit innate immune signals in the cells of the epidermis. For instance, eotaxin and eotaxin-3, important chemoattractants for eosinophils, are not only produced by fibroblasts, but the latter also by keratinocytes upon IL-4 stimulus (30).

We report here that percutaneous sensitization with recombinant HDM allergens Der p 1 and Der p 2 in the absence of any adjuvant causes eczema in BALB/c mice. Based on our data, we propose that the presented protocol renders a phenotype close to human allergic/atopic eczema and might represent a suitable experimental model for investigation of mechanisms of AE and novel treatment options.

## Notes

Figure S1 (a) BALB/c mice were wet-shaved and sensitized percutaneously with rDer p 1, rDer p 2 or PBS. For the first 8 weeks, 10-μg allergen was used per application. During the last two weeks of the immunisation, the allergen dose was increased to 100 μg/application. Application intervals and allergen dose are indicated on top, dates for blood sampling below the timeline. (b) In a second set of experiment, BALB/c mice (group size *n* = 8) were after wet-shaving sensitized percutaneously every third week, altogether 3 times, with the elevated concentration (100 μg) of rDer p 1 or rDer p 2. An additional group of mice was percutaneously pretreated with rDer p 1 (100 μg) and 30 min later with rDer p 2 (100 μg).

## References

[b1] Oyoshi MK, He R, Kumar L (2009). Adv Immunol.

[b2] Weber AS, Haidinger G (2010). Pediatr Allergy Immunol.

[b3] Akdis CA, Akdis M, Bieber T (2006). J Allergy Clin Immunol.

[b4] Bieber T (2010). Ann Dermatol.

[b5] Bieber T (2008). N Engl J Med.

[b6] Novak N, Bieber T, Leung DY (2003). J Allergy Clin Immunol.

[b7] Novak N, Gros E, Bieber T (2010). Clin Exp Immunol.

[b8] Dorner T, Rieder A, Lawrence K (2006). http://www.alternmitzukunft.at/upload/3006_AMZ_Allergiebericht.pdf.

[b9] de Benedictis FM, Franceschini F, Hill D (2009). Allergy.

[b10] Thomas WR, Smith WA, Hales BJ (2002). Int Arch Allergy Immunol.

[b11] Meyer CH, Bond JF, Chen MS (1994). Clin Exp Allergy.

[b12] Zock JP, Heinrich J, Jarvis D (2006). J Allergy Clin Immunol.

[b13] Szalai K, Fuhrmann J, Pavkov T (2008). Mol Immunol.

[b14] Matsuoka H, Maki N, Yoshida S (2003). Allergy.

[b15] de Halleux S, Stura E, VanderElst L (2006). J Allergy Clin Immunol.

[b16] Zhang J, Saint-Remy J-M, Garrod DR (2009). Allergy.

[b17] Elkhal A, Pichavant M, He R (2006). J Allergy Clin Immunol.

[b18] Yamamoto M, Haruna T, Yasui K (2007). Allergol Int.

[b19] Kawakami Y, Yumoto K, Kawakami T (2007). Allergol Int.

[b20] Huang CH, Kuo IC, Xu H (2003). J Invest Dermatol.

[b21] Matsuda H, Watanabe N, Geba GP (1997). Int Immunol.

[b22] Orita K, Hiramoto K, Inoue R (2010). Exp Dermatol.

[b23] Choi SE, Jeong MS, Kang MJ (2010). Exp Dermatol.

[b24] Yasue M, Yokota T, Suko M (1998). Lab Anim Sci.

[b25] Chapman MD, Wunschmann S, Pomes A (2007). Curr Allergy Asthma Rep.

[b26] Jeong SK, Kim HJ, Youm JK (2008). J Invest Dermatol.

[b27] Trompette A, Divanovic S, Visintin A (2009). Nature.

[b28] Österlundd C, Grönlund H, Polovic N (2009). Clin Exp Allergy.

[b29] Swamy M, Jamora C, Havran W (2010). Nat Immunol.

[b30] Igawa K, Satoh T, Hirashima M (2006). Allergy.

