# Supplementary figures and images for: Establishing an allergic eczema model employing recombinant house dust mite allergens Der p 1 and Der p 2 in BALB/c mice
Source: Exp Dermatol. 2012 Oct 15;21(11):842–6. doi: 10.1111/exd.12015 (PMC3532600; doi:10.1111/exd.12015)

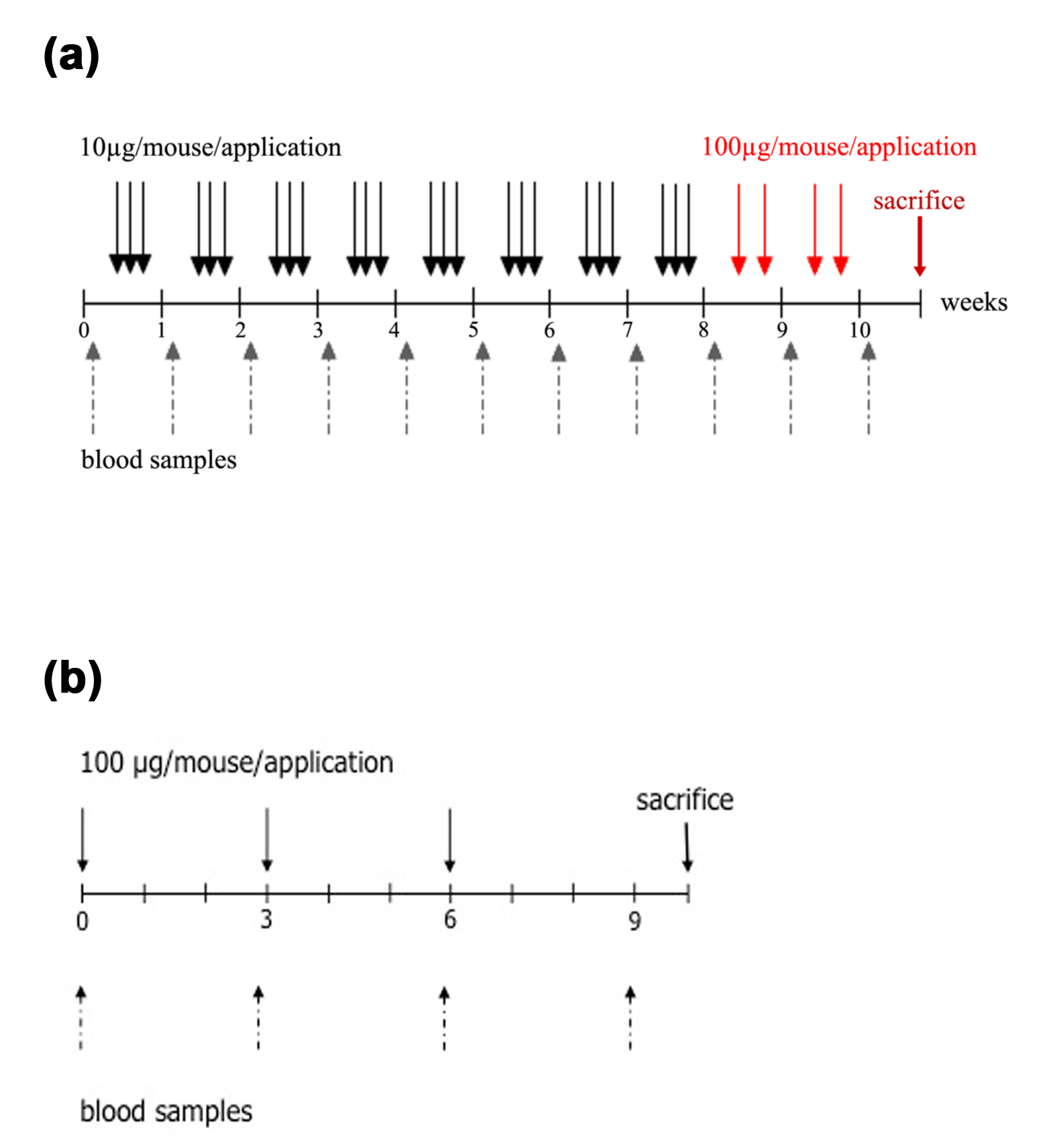

Supplement: Figure S1 — (a) BALB/c mice were wet-shaved and sensitized percutaneously with rDer p 1, rDer p 2 or PBS. For the first 8 weeks, 10-μg allergen was used per application. During the last two weeks of the immunisation, the allergen dose was increased to 100 μg/application. Application intervals and allergen dose are indicated on top, dates for blood sampling below the timeline. (b) In a second set of experiment, BALB/c mice (group size n = 8) were after wet-shaving sensitized percutaneously every third week, altogether 3 times, with the elevated concentration (100 μg) of rDer p 1 or rDer p 2. An additional group of mice was percutaneously pretreated with rDer p 1 (100 μg) and 30 min later with rDer p 2 (100 μg). [file exd0021-0842-SD1.tif]
